# Structural and perfusion magnetic resonance imaging of congenital lung malformations

**DOI:** 10.1007/s00247-020-04658-5

**Published:** 2020-04-17

**Authors:** Christian J. Kellenberger, Christina Amaxopoulou, Ueli Moehrlen, Peter K. Bode, Andreas Jung, Julia Geiger

**Affiliations:** 1 Department of Diagnostic Imaging, University Children’s Hospital Zürich, Steinwiesstr. 75, CH 8032 Zürich, Switzerland; 2 Children’s Research Centre, University Children’s Hospital Zürich, Zürich, Switzerland; 3 Department of Pediatric Surgery, University Children’s Hospital Zürich, Zürich, Switzerland; 4 Institute of Pathology, University Hospital Zürich, University of Zürich, Zürich, Switzerland; 5 Division of Pulmonology, University Children’s Hospital Zürich, Zürich, Switzerland

**Keywords:** Bronchopulmonary malformation, Children, Lung, Magnetic resonance imaging, Perfusion imaging

## Abstract

**Background:**

A radiation-free advanced imaging modality is desirable for investigating congenital thoracic malformations in young children.

**Objective:**

To describe magnetic resonance imaging (MRI) findings of congenital bronchopulmonary foregut malformations and investigate the ability of lung MRI for their classification.

**Materials and methods:**

This is a retrospective analysis of consecutive MRI examinations performed for suspected congenital lung anomalies in 39 children (median age: 3.8 months, range: 2 days–15 years). Morphological and perfusion findings were characterised on respiratory-gated fast spin echo and dynamic contrast-enhanced sequences obtained at 1.5 tesla. Abnormalities were classified independently by two readers and compared to an expert diagnosis based on pathology, surgery and/or other imaging.

**Results:**

Main diagnoses included bronchopulmonary lesions in 33 patients, scimitar syndrome in 4 patients, pulmonary arteriovenous malformation and oesophageal duplication cyst in one patient each. Of 46 observed abnormalities, 44 (96%) were classified correctly with very good interobserver agreement (96% concordance rate). The 39 detected lung lesions included isolated overinflation (17/39, 44%), cystic pulmonary airway malformation (8/39, 21%), bronchopulmonary sequestration (7/39, 18%), bronchogenic cyst (4/39, 10%) and hybrid lesion (3/39, 8%). All lung lesions presented as perfusion defect at peak pulmonary enhancement. Non-cystic lesions showed a delayed peak (median delay: 2.8 s, interquartile range: 0.5 to 4.0 s) in relation to normal lung parenchyma.

**Conclusion:**

A dedicated lung MRI protocol including respiratory compensated sequences, dynamic angiography and perfusion is able to reliably delineate parenchymal and vascular components of congenital bronchopulmonary foregut malformations. Therefore, MRI may be considered for comprehensive postnatal evaluation of congenital thoracic malformations.

## Introduction

Congenital lung disease includes a broad variety of rare developmental anomalies that may be clinically relevant [1, 2], including congenital pulmonary airway malformation (CPAM), bronchopulmonary sequestration, bronchogenic cyst, congenital segmental or lobar overinflation, venolobar syndrome and other vascular malformations. With the advent of prenatal ultrasound (US), congenital lung lesions have been estimated at about 1 in 3,000 live births [3]. Imaging plays an eminent role in lesion detection and characterisation, which are the basis for antenatal and postnatal management of affected foetuses and infants. Many congenital thoracic malformations are considered a spectrum of an in utero airway obstruction--lung malformation sequence with accompanying vascular anomalies [1, 2]. Systematic evaluation of all components (airway, lung parenchyma and vasculature) is mandatory for lesion delineation and classification [4]. Accurate diagnosis and localisation of the different entities may influence patient management or provide important information for surgery.

During pregnancy, US and magnetic resonance imaging (MRI) are commonly used to monitor lung lesions [5–10], while postnatal assessment usually relies on chest radiography and computed tomography (CT) [2, 11, 12]. As prenatal evaluation and chest radiography are not very reliable for fully defining the type of lesion [1, 2, 13], and because lesions that apparently disappear during pregnancy may still be detectable after birth [2, 13], it is general practice to investigate affected children by contrast-enhanced CT postnatally before 6 weeks of age or between 3 and 12 months [6, 14, 15]. Multi-detector chest CT angiography has been suggested for assessing potential extrapulmonary blood supply [11, 14].

The main disadvantage of CT in the paediatric population is ionising radiation, which can still be considered significant even when ALARA (as low as reasonably achievable) principles and iterative reconstruction techniques are applied, resulting in estimated effective doses of about 1–2 mSv for an infant [12, 16]. Serial CT, which has been recommended by some centres for following asymptomatic lesions for up to 10 years [6, 17, 18], adds to the radiation burden. Therefore, a radiation-free technique is desirable for diagnosing and monitoring congenital lung lesions. Although MRI has been advocated for assessing solid and vascular components of congenital lung lesions, it is generally not considered a first-line modality for characterising lung parenchymal abnormalities [11, 14, 19, 20].

Since the introduction of a dedicated lung MRI protocol at our institution in 2013, we have increasingly imaged children with congenital lung abnormalities. The aim of this work is to describe the MRI findings of congenital bronchopulmonary foregut malformations and investigate the diagnostic performance of MRI for their classification.

## Materials and methods

### Patients

In this retrospective study, lung MRI studies performed to assess congenital lung lesions from July 2013 to July 2019 were searched on the picture archiving and communication system (PACS) of our tertiary university paediatric hospital. All available imaging studies and electronic medical records were reviewed for the 39 consecutive patients identified. During the same 6-year period, 335 lung MRI examinations were performed at our institution.

Detection and classification of the lung lesions on MRI were compared to a final diagnosis reached by an expert consensus panel (C.J.K., a radiologist with 21 years of experience in paediatric chest imaging and MRI; P.K.B., a paediatric pathologist with 16 years of experience; and U.M., a surgeon with 15 years of experience in paediatric general and thoracic surgery) based on surgery, pathology, other imaging (including prenatal and follow-up studies) and/or clinical follow-up. The MRI morphology and perfusion of the congenital abnormalities were described. The included patients and/or their parents had given consent to retrospective data analysis. The study was approved by the responsible governmental ethics committee. As part of the clinical hospital routine, patients and/or parents consented to MRI with contrast administration and sedation when applicable.

### Lung magnetic resonance imaging

All MRI scans were performed on a 1.5-T scanner (Discovery MR 450; GE Healthcare, Waukesha, WI) with a multi-array flexible surface coil covering the chest. Most of the children (37/39, 94.9%) were imaged under sedation with propofol (*n*=27), chloral hydrate (*n*=2) or general anaesthesia with intubation (*n*=8) applied by anaesthesiologists according to hospital routine.

As described previously [21], the routine protocol included fast imaging employing steady-state acquisition (FIESTA) in three orthogonal planes, axial and coronal partial Fourier acquisition single-shot fast spin echo (SSFSE), axial respiratory-gated T2-weighted fast spin echo sequence with periodically rotated overlapping parallel lines with enhanced reconstruction (PROPELLER), coronal respiratory-gated proton-density weighted fast spin echo sequence (FSE), as well as axial and coronal T1-weighted fast spin echo or fast spoiled gradient echo acquisition in the steady state (FSPGR) before and after intravenous contrast injection. Perfusion imaging was performed with a dynamic contrast-enhanced three-dimensional gradient echo sequence (time-resolved imaging of contrast kinetics [TRICKS angiography]). By under-sampling the peripheral k-space and parallel imaging, we achieved a temporal resolution of 1–2 s. Image acquisition was started with intravenous injection of a short bolus of gadolinium-based contrast agent (0.1 mmol/kg body weight; gadoteric acid; [Dotarem; Guerbet AG, Zürich, Switzerland]) and comprised 40 phases (duration: 40–80 s). Typical sequence parameters for imaging the lung in an infant are given in Table 1. The time for the MRI study, including time for sedation, ranged from 30 to 40 min; the mean imaging time for acquiring all MRI sequences was about 20 min.

**Table 1:** Lung magnetic resonance imaging protocol for infants

Sequence	FIESTA	SSFSE	T2-PROPELLER	PD-FSE	TRICKS	T1-PROPELLER
Imaging planes	Axial, coronal, sagittal	Axial, coronal, sagittal	Axial	Coronal	Coronal	Axial
Repetition time (ms)	3.5	280	2,500	2,500	3.1	570
Echo time (ms)	1.5	34	46	11	1.3	10
Flip angle (°)	45	90	140	90	25	90
Echo train length	na	ss	16	30	na	6
Number of excitations	1	hF	2.6	4	hF	3
Fat saturation	no	no	yes	no	no	yes
Respiratory gating	no	no	yes	yes	no	no
Slice (mm)	4	4	4	4	2.5	4
Field of view (cm)	27	27	22	22	22	22
Matrix	160×256	288×192	288×288	352×256	256×160	320×320
Imaging time	3×20 s	3×10 s	4 min	3 min	40 s	2×5 min

*FIESTA* fast imaging employing steady-state acquisition, *hF* half-Fourier acquisition, *na* not applicable, *PD-FSE* proton density weighted fast spin echo, *ss* single shot, *SSFSE* half-acquisition single-shot fast spin echo, *T1-PROPELLER* T1-weighted periodically rotated overlapping parallel lines with enhanced reconstruction obtained after contrast administration, *T2-PROPELLER* T2-weighted periodically rotated overlapping parallel lines with enhanced reconstruction, *TRICKS* time-resolved imaging of contrast kinetics

### Image evaluation and lesion classification

All lung MRI examinations were reviewed separately on a PACS workstation (IDS7; Sectra Medical Systems, Linköping, Sweden) by two paediatric radiologists (J.G., 10 years’ experience, and C.J.K.) to assess inter-reader agreement for detection and classification of the lung lesions. Discrepant results were resolved in a consensus reading. To assess intra-reader agreement and agreement of MRI with CT, one reader (C.J.K.) performed a formal reading of the available CT studies and repeated the MRI assessment after an interval of 5 months.

On MRI, morphological assessment of the lung parenchyma and mediastinum relied mainly on the respiratory gated fast spin echo sequences. Abnormalities of the vasculature and lung perfusion were sought on the contrast-enhanced dynamic series.

The location and characteristics of the detected lesions were described. The lesions were categorised as cystic filled with air or fluid, and consolidated or overinflated (decreased parenchymal intensity without discernible cysts). The size and number of cysts were noted. One observer (C.J.K.) measured the size of the lung lesions on both CT and MRI at two separate readings.

Structures with high signal intensity on T2-weighted images within the lesions were noted and interpreted as mucus-filled bronchial structures. Systemic arterial supply and anomalous venous drainage were sought on angiographic images from the dynamic contrast-enhanced series. Perfusion of the lung lesions was qualitatively assessed on parametric images (subtraction images at signal peak enhancement of the lungs, and enhancement integral representing pulmonary blood volume) that were constructed with commercially available software (Volume Viewer, AW Server 3.2; GE Healthcare). Perfusion of the lesion was rated in comparison to unaffected lung parenchyma as absent, decreased, normal or increased. Timing of peak enhancement of the lesion was compared to that of surrounding lung parenchyma and rated as normal or delayed.

One reader (C.J.K.) measured average signal intensity at peak enhancement and signal enhancement integral for all lung lesions and corresponding normal lung parenchyma with a fixed size region of interest (ROI; area range: 0.5 to 1 cm^2^) per patient. The time interval between peak enhancement of the lung lesion and normal parenchyma was noted. Ratios for signal enhancement integral were calculated between lesions and normal lung parenchyma.

**Fig. 1:**
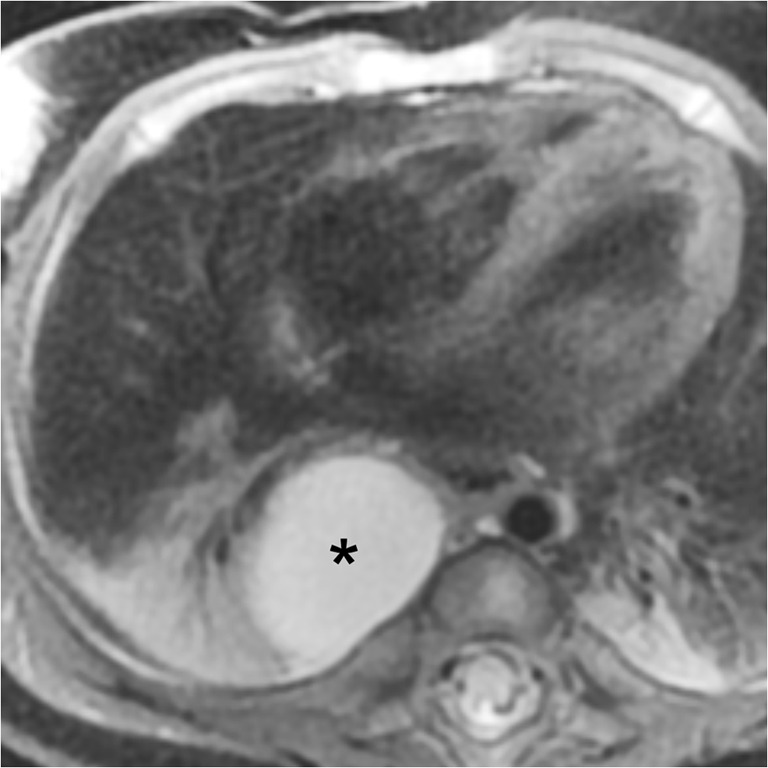
Oesophageal duplication cyst in a 3-month-old girl with foetal ultrasound diagnosis of cystic congenital pulmonary airway malformation. Axial fat-saturated T2-weighted image shows broad connection of the unilocular fluid-filled cyst (*) with the oesophageal wall

**Fig. 2:**
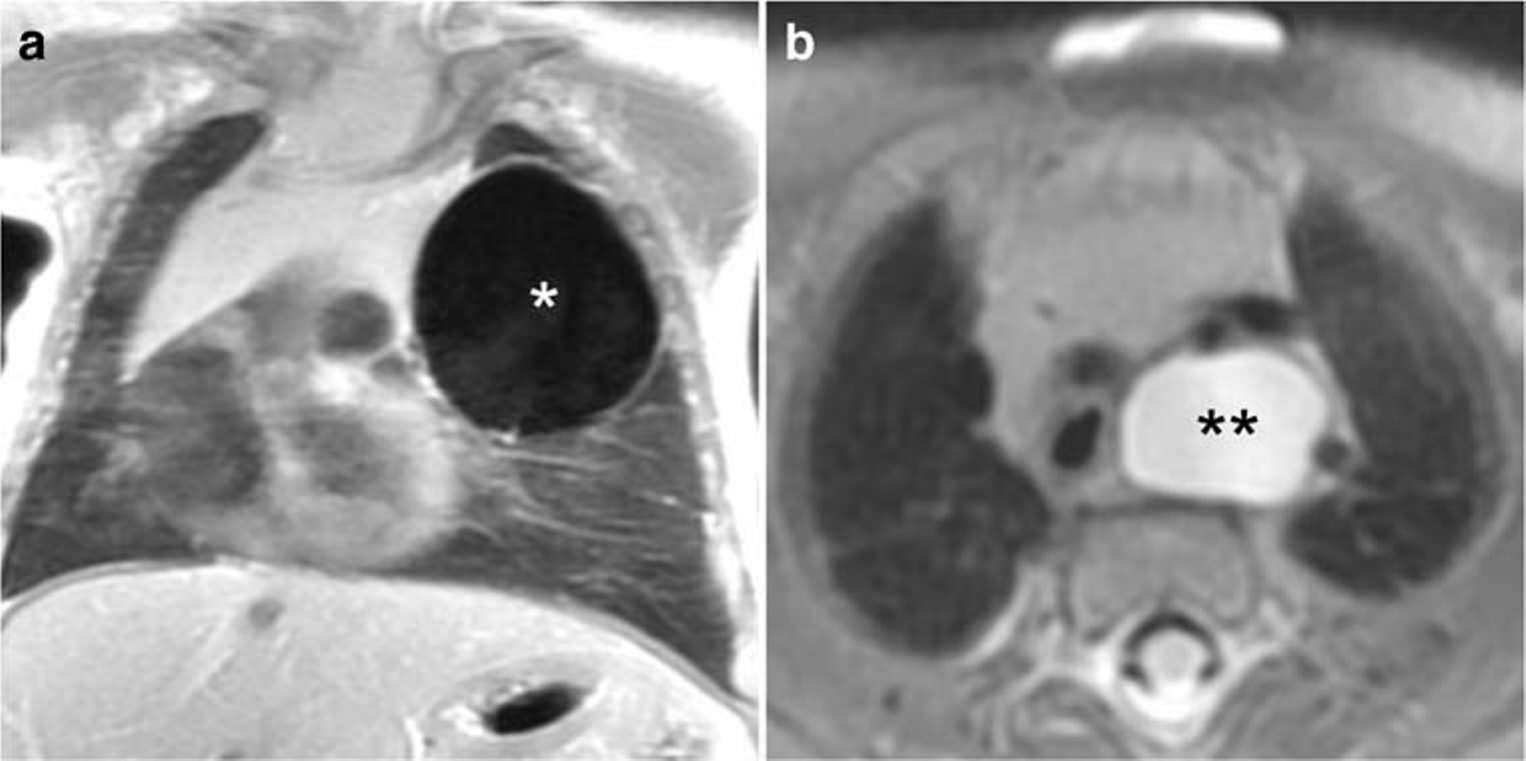
Bronchogenic cysts in an 8-week-old boy. **a** Coronal proton density MR image shows a single air-filled cyst (*) in the left upper lobe following thoraco-amniotic drainage in utero. **b** Axial T2-weighted image shows a second mediastinal fluid-filled cyst (**) with connection to the tracheal wall

**Fig. 3:**
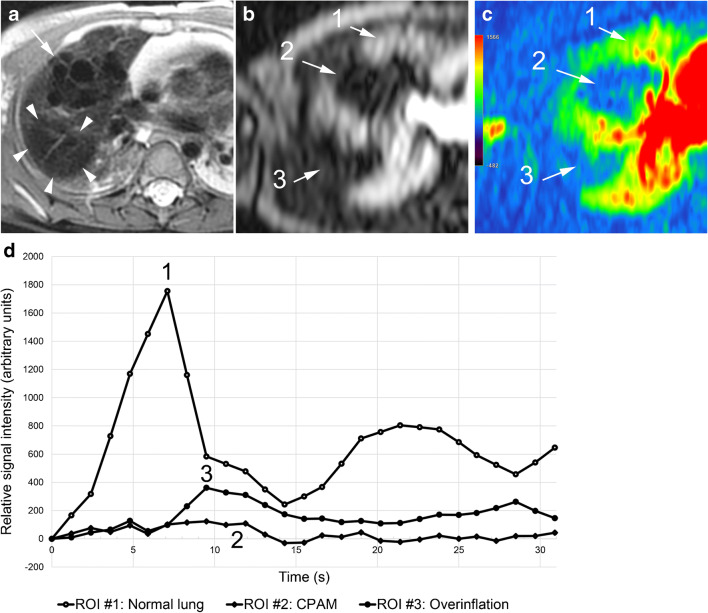
Cystic congenital pulmonary airway malformation (CPAM) type 2 and isolated overinflation in an 8-month-old girl. **a** Axial T2-weighted image shows multiple air-filled cysts (*arrow*) in the anterior upper lobe segment and overinflation of the posterior upper lobe segment (*arrowheads*). **b, c** Axial image at peak enhancement (**b)** and parametric map (enhancement integral) (**c**) show the CPAM (2 in **b, c**) and isolated overinflation (3 in **b, c**) as perfusion defects. **d** Signal intensity – time curves show minimal perfusion of the CPAM (2 in **d**) and delayed enhancement of the overinflation (3 in **d**) in comparison to normal lung parenchyma (1 in **b, c** and **d**). *ROI* region of interest

**Fig. 4:**
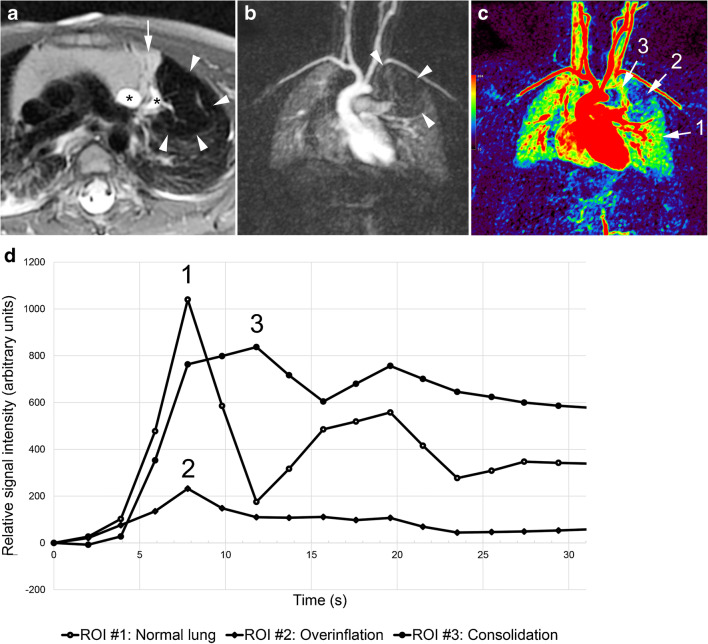
Isolated overinflation (bronchial atresia) in an 11-month-old boy. **a** Axial T2-weighted image shows a hypointense lung area (*arrowheads*) with rarefication of the pulmonary markings, central fluid-filled round and linear bronchial structures (*, mucoceles), and some consolidation (*arrow*). **b** Coronal contrast-enhanced image at pulmonary peak enhancement shows the pulmonary lesion as a perfusion defect (*arrowheads*). **c** Coronal parametric map (enhancement integral). **d** Signal intensity – time curves. There is decreased perfusion of the overinflated area with pulmonary arterial peak (2 in **c** and **d**), while the consolidation (3 in **c** and **d**) shows delayed peak enhancement when compared to normal lung parenchyma (1 in **c** and **d**). *ROI* region of interest

**Fig. 5:**
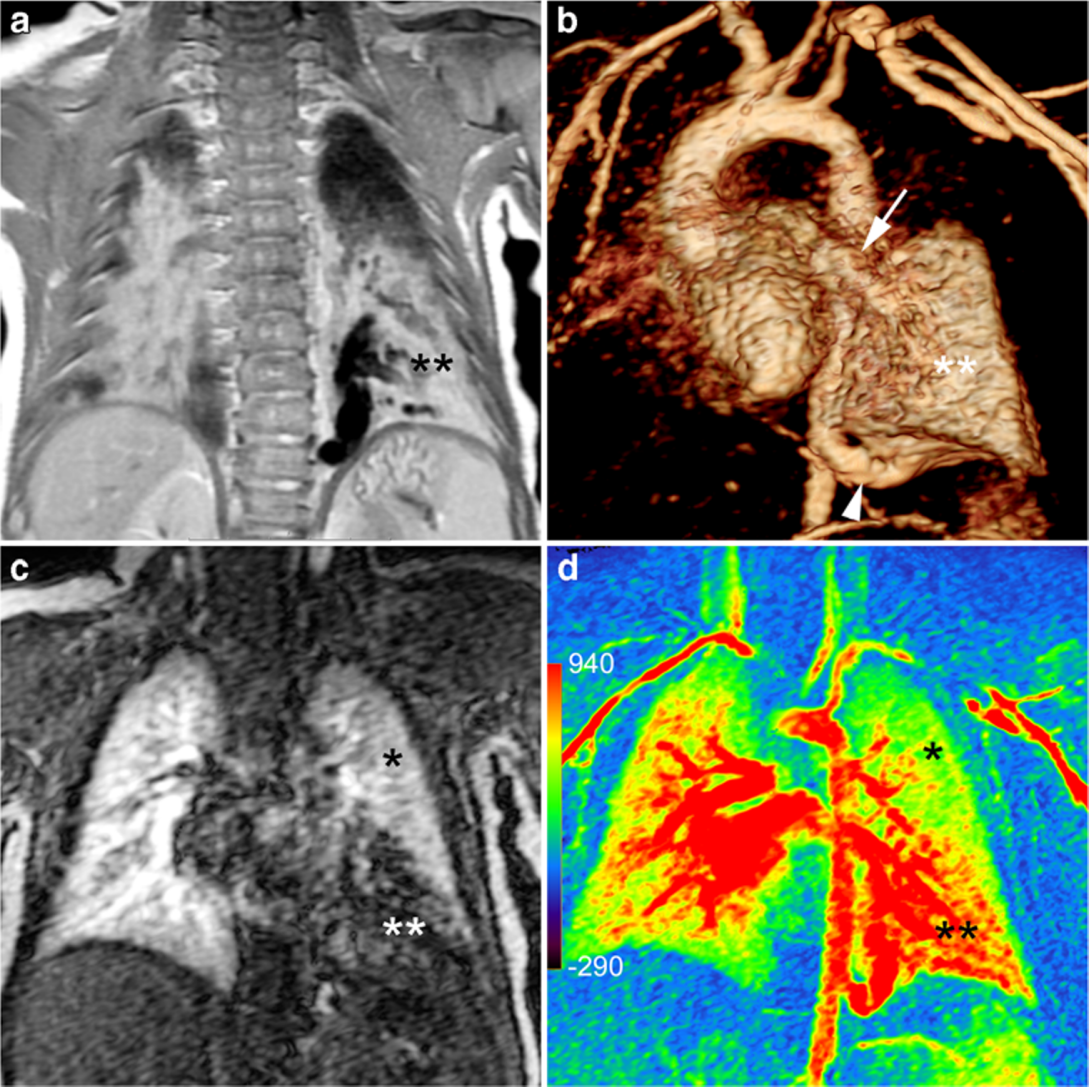
Bronchopulmonary sequestration in a 3-month-old boy. **a** Coronal proton density MR image shows consolidation in the left lower lobe (**) and prominent vessels with flow voids. **b** Left anterior oblique volume rendered angiographic view shows the lung lesion (**), its systemic arterial supply (*arrowhead*) from the descending aorta and venous drainage (*arrow*) to the left lower pulmonary vein. **c**, **d** Coronal image at peak pulmonary enhancement (**c**) shows a perfusion defect (**) and parametric map (enhancement integral) (**d**) shows increased perfusion (**) compared to normal lung (* in **c** and **d**)

Based on location, morphological and perfusion findings, the observed abnormalities were classified into nine categories, including different foregut cysts, lesions with pulmonary overinflation, cystic pulmonary malformations, and predominantly vascular lesions and scimitar syndrome. The designations, synonyms, pathology and imaging findings of the different entities diagnosed in our cohort are detailed in Table 2 and illustrated with Figs. 1, 2, 3, 4 and 5.

**Table 2:** Classification of congenital bronchopulmonary, lung and vascular malformations in 39 patients with magnetic resonance imaging

Entity (synonyms)	Pathology	Imaging findings (MRI and CT)
Bronchogenic cyst	Fluid-filled cyst lined by respiratory type epithelium with underlying fascicles of smooth muscle and mature cartilage	Solitary unilocular cyst filled with fluid/mucus adjacent to central airways or in the periphery of the lung
Oesophageal duplication cyst	Fluid-filled cyst attached to oesophagus and covered by 2 muscle layers	Solitary fluid-filled cyst bordering the oesophagus
Isolated overinflation (isolated bronchial atresia, segmental or lobar emphysema/hyperinflation/overinflation)	Obstruction to airways at different levels Normal lung parenchyma with airspace enlargement	Lobar, segmental or subsegmental area with decreased signal intensity or attenuation, ±mass effect or architectural distortion, ±fluid-filled dilated bronchial structures (mucocele, linear hyperintensities)
Overinflation with systemic feeding artery (bronchopulmonary sequestration)	+systemic artery to lesion	Findings of overinflation or consolidation, and systemic artery supplying the lesion
Overinflation with systemic feeding artery and cysts (hybrid lesion: bronchopulmonary sequestration with parenchymal cysts)	+systemic artery to lesion +multiple small cysts (CPAM type 2)	Findings of overinflation or consolidation, systemic artery supplying the lesion and identifiable parenchymal air-filled cysts (<2 cm)
Cystic congenital pulmonary airway malformation (congenital cystic adenomatoid malformation [CCAM], congenital pulmonary airway malformation [CPAM])	CPAM type 1 (large cyst lesion) CPAM type 2 (small cyst lesion)	Single predominant or multiple air-filled or air/fluid-filled cysts (largest cyst >2 cm) Multiple small cysts (<2 cm) ±solid or hyperinflated lung areas
Systemic arterial supply to normal lung (major aortopulmonary collateral arteries [MAPCA])		Systemic arterial supply to normally aerated lung parenchyma
Pulmonary arteriovenous malformation		Anomalous connection between peripheral pulmonary artery and vein
Scimitar syndrome (venolobar syndrome, hypogenetic lung syndrome)		Hypoplastic right lung with two lobes Hypoplastic right pulmonary artery Partial anomalous venous drainage ±systemic arterial supply to lower lobe ±diaphragmatic hernia, horseshoe lung

### Statistical analysis

Descriptive data were checked with the Shapiro-Wilk W test for normal distribution. Data with normal distribution are given as mean±standard deviation (SD). Data without normal distribution are given as median and interquartile range (IQR). Frequencies are given as fraction and percentage. Agreement between MRI and final expert diagnosis, agreement between MRI and CT in a subgroup of patients with both modalities available, intra-reader agreement and inter-reader agreement for classifying the congenital abnormalities on MRI were described with concordance rates. Perfusion measures were compared between lesions and normal lung parenchyma with a paired samples *t*-test and between different lesions with an independent samples *t*-test. The statistical analysis was performed with MedCalc Statistical Software version 19.0.5 (MedCalc Software Ltd., Ostend, Belgium). A *P*-value <0.05 was considered significant.

## Results

### Patients

The study population consisted of 39 children, 18 girls and 21 boys, with a median age of 3.8 months, interquartile range of 3.1 to 12.1 months and age range of 2 days to 14.7 years at MRI examination.

### Diagnostic performance of MRI

In Table 3, the clinical presentation, MRI findings and final expert diagnosis are detailed for all patients and lesions. The final diagnosis was supported by pathology in 24/39 (61.5%) patients and 28/46 (60.9%) abnormalities. In another 3/39 (7.7%) patients and 3/46 (6.5%) abnormalities, the findings were confirmed at surgery. In the remaining 12/39 (30.8%) patients and 15/46 (32.6%) abnormalities, the final diagnosis was confirmed by other imaging.

**Table 3:** MRI findings and diagnoses of 46 abnormalities in 39 patients with congenital bronchopulmonary foregut malformations

Case #	Final diagnosis	Presentation	Patient age	MRI findings	Location	Proof of diagnosis	Management
11	Bronchogenic cyst	Incidental finding	15 years	Unilocular fluid-filled cyst	Subcarinal	Pathology	Cyst resection
31	Bronchogenic cyst	Incidental finding	3 years	Unilocular fluid-filled cyst	Paratracheal	CXR	Conservative
32	Bronchogenic cyst (with drainage in utero)	Prenatal diagnosis	4 months	Large air-filled cyst	LUL	Pathology	Lobectomy
32	Bronchogenic cyst	Incidental finding	4 months	Fluid-filled cyst	Paratracheal	Pathology	Cyst resection
36	Oesophageal duplication cyst	Prenatal diagnosis	3 months	Fluid-filled cyst	Paravertebral	Pathology	Cyst resection
12	Bilobar overinflation (atresia intermediate bronchus)	Prenatal diagnosis	2 days	Air-filled cysts surrounded by fluid-filled lung parenchyma	ML, RLL	Pathology	Bilobectomy
7	Lobar overinflation	Airway infection	9 months	Lobar overinflation	LUL	Pathology	Lobectomy
25	Lobar overinflation	Prenatal diagnosis	3 months	Lobar overinflation	LUL	Foetal US, follow-up MRI	Conservative
27	Lobar overinflation	Respiratory distress	13 months	Lobar overinflation	LUL	Follow-up CXR	Conservative
1	Segmental overinflation (bronchial atresia)	Prenatal diagnosis	11 months	Areas of consolidation and overinflation, mucocele	LUL	Pathology	Lobectomy
9	Segmental overinflation	Prenatal diagnosis	8 months	Segmental overinflation	RUL	Pathology, CT	Lobectomy
9	Segmental overinflation	Prenatal diagnosis	8 months	Segmental overinflation	ML	Pathology, CT	Lobectomy
20	Segmental overinflation	Incidental finding	3 days	Partly fluid- and air-filled lung segment	RLL	Follow-up MRI, CT	Conservative
20	Segmental overinflation	Incidental finding	3 days	Partly fluid- and air-filled lung segment	LLL	Follow-up MRI, CT	Conservative
24	Segmental overinflation	Prenatal diagnosis	4 months	Segmental overinflation	RLL	Foetal US	Conservative
26	Segmental overinflation	Respiratory distress	7 months	Segmental overinflation	LUL	Follow-up CXR	Conservative
37	Segmental overinflation	Prenatal diagnosis	14 months	Segmental overinflation	LLL	Foetal US and MRI	Conservative
8	Subsegmental overinflation	Prenatal diagnosis	2 months	Architectural distortion, small consolidation	RLL	Pathology	Lobectomy
10	Subsegmental overinflation	Prenatal diagnosis	7 months	Subsegmental overinflation	RUL	Foetal US and MRI	Conservative
29	Subsegmental overinflation	Incidental finding	13 months	Subsegmental overinflation	RUL	CT, follow-up MRI	Conservative
37	Subsegmental overinflation	Prenatal diagnosis	14 months	Subsegmental overinflation	LUL	Foetal US and MRI	Conservative
3	Intralobar sequestration	Prenatal diagnosis	4 months	Segmental overinflation, systemic artery	RLL	Pathology, CT	Lobectomy
6	Intralobar sequestration	Prenatal diagnosis	4 months	Consolidation, systemic artery	RLL	Pathology	Lobectomy
13	Intralobar sequestration	Prenatal diagnosis	3 months	Consolidation, systemic arteries	RLL	Pathology	Lobectomy
30	Intralobar sequestration	Prenatal diagnosis	6 days	Consolidation containing small fluid-filled structures, systemic artery	RLL	Pathology	Lobectomy
33	Intralobar sequestration	Prenatal diagnosis	3 months	Consolidation, systemic artery	LLL	Pathology	Segment resection
38	Intralobar sequestration	Prenatal diagnosis	3 months	Consolidation, systemic artery	LLL	Pathology	Lobectomy
13	Extralobar sequestration	Prenatal diagnosis	3 months	Consolidation, systemic arteries, contralateral pulmonary venous drainage	LLL	Pathology	Lobectomy
15	Hybrid lesion	Recurrent pneumonia	4 years	Small air-filled cysts, consolidation, systemic artery	LLL	Pathology, CT	Lobectomy
17	Hybrid lesion	Prenatal diagnosis	5 months	Small air-filled cysts, consolidation, systemic artery	RLL	Foetal US and MRI, follow-up MRI	Conservative
39	Hybrid lesion	Prenatal diagnosis	3 months	Small air-filled cysts, systemic artery	LLL	Pathology	Lobectomy
4	Cystic CPAM type 1	Prenatal diagnosis	3 months	Large air-filled cysts	LUL	Pathology	Lobectomy
5	Cystic CPAM type 1	Incidental finding	12 years	Large air-filled cysts	LLL	Pathology, CT	Lobectomy
14	Cystic CPAM type 1	Prenatal diagnosis	4 months	Large air-filled cysts	RLL	Pathology	Lobectomy
23	Cystic CPAM type 1	Prenatal diagnosis	4 months	Large air-filled cysts	LUL	Pathology, CT	Lobectomy
35	Cystic CPAM type 1	Incidental finding	15 months	Large air-filled cyst	LLL	Pathology	Lobectomy
9	Cystic CPAM type 2	Prenatal diagnosis	8 months	Small air-filled cysts	RUL	Pathology, CT	Lobectomy
16	Cystic CPAM type 2	Prenatal diagnosis	2 months	Small air-filled cysts, systemic artery*	RLL	Pathology	Lobectomy
19	Cystic CPAM type 2	Prenatal diagnosis	4 months	Small air-filled cysts	ML	Pathology, CT	Lobectomy
21	Cystic CPAM type 2	Prenatal diagnosis	3 months	Small air-filled cysts	RUL	Pathology	Lobectomy
20	MAPCAs	Prenatal diagnosis	3 days	Systemic arteries (direct MAPCAs)	RUL, LLL	Surgery	Total repair**
2	Scimitar syndrome	Incidental finding	30 months	Hypoplastic right lung, PAPVR, systemic artery	Right lung	Surgery, angiography, CT	PAPVR repair
18	Scimitar syndrome	Recurrent airway infection	25 months	Hypoplastic right lung, PAPVR, systemic artery	Right lung	Echocardiography	Conservative
28	Scimitar syndrome	Incidental finding	2 months	Hypoplastic right lung, PAPVR, systemic artery	Right lung	Echocardiography	Conservative
22	Scimitar syndrome	Respiratory distress	6 days	Hypoplastic right lung, PAPVR, systemic arteries	Right lung	Surgery, angiography, CT	PDA closure, PAPVR repair
34	Pulmonary AVM	Hypoxemia	6 days	Enhancing mass lesion with pulmonary arterial and venous connection	RLL	Angiography	Embolisation

*AVM* arteriovenous malformation, *CPAM* congenital pulmonary airway malformation, *CXR* chest radiograph, *LLL* left lower lobe, *LUL* left upper lobe, *MAPCA* major aortopulmonary collateral artery, *ML* middle lobe, *PAPVR* partial anomalous pulmonary venous return, *PDA* patent ductus arteriosus,

*RUL* right upper lobe, *RLL* right lower lobe

* Phrenic artery misinterpreted as systemic arterial supply to lung

** Pulmonary atresia repair with right ventricle to pulmonary artery conduit and unifocalisation of pulmonary arteries

The indication for MRI was prenatal detection of a lung lesion in 25/39 (64.1%) patients, an incidental imaging finding in 7/39 (17.9%) patients and respiratory symptoms in 7/39 (17.9%) patients. The main MRI diagnoses were a congenital bronchopulmonary lesion in 33/39 (84.6%) patients, scimitar syndrome in 4/39 (10.3%) patients, pulmonary arteriovenous malformation in 1/39 (2.6%) and oesophageal duplication cyst (Fig. 1) in 1/39 (2.6%) patients. Overall, 44/46 (95.7%, 95% confidence interval [CI] 85.2% to 99.5%) abnormalities were correctly diagnosed (Table 3).

In the 33 patients with congenital bronchopulmonary abnormalities, 39 lung lesions were detected, including isolated overinflation (17/39, 43.6%), cystic congenital pulmonary airway malformations (8/39, 20.5%), bronchopulmonary sequestrations (7/39, 17.9%), bronchogenic cysts (4/39, 10.3%) and hybrid lesions (3/39, 7.7%). These bronchopulmonary abnormalities were correctly classified on MRI in 37/39 cases (94.9%, 95% CI 82.7% to 99.4%). An isolated bronchial atresia was misdiagnosed as cystic pulmonary airway malformation because dilated segmental bronchi were partially filled with fluid and air resembling air filled cysts (Case 12). An isolated overinflation with small cysts was misdiagnosed as a hybrid lesion because a phrenic artery was mistaken as a systemic artery supplying the lung lesion (Case 16), which was not confirmed at surgery. In the subgroup of 24 patients with resected lung lesions (28 lesions), the concordance rate between MRI and pathology was 26/28 (92.9%, 95% CI 76.5% to 99.1%) for lesion diagnosis and detection of systemic arterial supply.

Intra-reader and inter-reader agreement for classifying all 46 detected congenital abnormalities listed in Table 3 was high with concordance rates of 97.8% (95% CI 88.5% to 99.9%) and 95.7% (95% CI 85.2% to 99.5%), respectively. One observer missed a systemic artery in a lung lesion and in another case misinterpreted the right phrenic artery as systemic arterial supply to the lung lesion. There was complete agreement between the readers for locating the lesions to lung lobes or mediastinum.

### Morphology and perfusion of the bronchopulmonary abnormalities (Table 3)

Bronchogenic cysts were defined as a single unilocular cyst filled with high signal intensity fluid (three cases). In one patient, a large bronchogenic cyst had been treated in utero with a thoraco-amniotic shunt and presented as a single air-filled cyst at 2 months of age (Fig. 2). Air-filled cysts (Fig. 3) of varying size and number were detected in cystic congenital pulmonary airway malformations (eight lesions) or hybrid lesions (three lesions). All cystic lesions (eight congenital pulmonary airway malformations, four bronchogenic cysts, three hybrid lesions and one oesophageal duplication cyst) showed no contrast enhancement within the cyst lumen.

Most of the lesions classified as isolated overinflation (14/17, 82.4%) (Figs. 3 and 4) presented as a hypointense lung area with architectural distortion (10/14, 24.4%) and small solid areas (5/14, 50%). Only 3/17 (17.6%) parenchymal areas of isolated overinflation, which were imaged during the first 3 days after birth, were filled with fluid showing homogenous high signal on T2-weighted images.

Most bronchopulmonary sequestrations (6/7, 85.7%) presented as consolidation (Fig. 5). Highly intense linear or branching structures were seen centrally in isolated overinflation (7/17, 41.2%) (Fig. 4), in bronchopulmonary sequestration (6/7, 85.7%) and in hybrid lesions (2/3, 66.7%).

The four lung areas with systemic perfusion in scimitar syndrome were hardly detectable on morphological sequences but were clearly evident as perfusion defects on images at peak lung enhancement.

All bronchopulmonary lesions presented as perfusion defect at peak lung enhancement (Figs. 3, 4 and 5). All isolated overinflation showed decreased peak enhancement, with a pulmonary arterial peak in 5/17 (29.4%) lesions and a delayed peak in 12/17 (70.6%). Sequestrations showed delayed and increased (4/7, 57.1%), similar (1/7, 14.3%) or decreased (2/7, 28.6%) peak enhancement compared to normal lung parenchyma. Lung lesions with detectable systemic arteries showed significantly higher enhancement ratios than those without a detectable systemic artery (mean±SD: 0.97±0.62 vs. 0.40±0.23, *P*=0.009, independent samples *t*-test) but no significant difference in the delay of peak enhancement (mean±SD: 2.9±1.2 s vs. 2.1±2.0 s, *P*=0.237, independent samples *t*-test). Median and interquartile range for the delay of peak enhancement was 2.8 s (0.5 to 4.0 s) for all non-cystic pulmonary lesions. Perfusion parameters for the different lung lesions are detailed in Table 4.

**Table 4:** Perfusion parameters of 31 congenital lung lesions and corresponding normal lung parenchyma

Entity		Signal enhancement integral (arbitrary units, mean±SD)		Enhancement ratio (median [IQR] or mean±SD)	Delay of peak enhancement (s, median [IQR] or mean±SD)
	*n*	Normal lung	Lesion		
Isolated overinflation	17	417±204	144±85*	0.4 (0.2–0.5)	2.1 (0.0–4.1)
Bronchopulmonary sequestration	7	385±297	415±306	1.3±0.6	3.1±0.7
Hybrid lesion	3	689±313	287±196	0.4±0.1	2.0, 2.2, 4.5
Lung area with systemic supply in scimitar syndrome	4	560±259	322±212	0.6±0.2	0.0, 3.4, 3.8, 3.9

*SD* standard deviation*, IQR* interquartile range

* Comparison between lesion and normal lung with paired *t*-test, *P*=0.0001

### Comparison between MRI and CT

A comparison of the MRI studies to CT examinations was available in 13/39 (33.3%) patients. In 7/13 (53.8%) patients, earlier CT studies had been performed (5/7, 71.4% at referring hospitals) and MRI was obtained as follow-up for surgical planning at our institution. In 6/13 (46.2%) patients, CT was performed after MRI: as preoperative follow-up for surgical planning in 4/6 (66.7%) cases and for assessing airway compression in 2 cases. On average, CT was performed 23 days (median) before MRI, ranging from 2 years before to 6 months after MRI.

In these 13 patients, the MRI and CT diagnosis and classification of 16 detected abnormalities were concordant in all cases. All (6/6, 100%) systemic arteries were detected both on CT and MRI. In 14 lung lesions, the volume of the lesions measured slightly smaller on MRI than on CT (mean difference–0.4 ml, limits of agreement–1.6 ml to 0.9 ml).

## Discussion

In the surgical, pathological and imaging literature, numerous terminologies are used to describe congenital pulmonary lesions. For this study, we formulated an imaging classification (Table 2) that takes into account the heterogeneous nature and characteristics of the diverse congenital malformations. Our classification was based on and modified from previous descriptions of pathological features by Stocker [22], Langston [1] and an imaging review by Newman [2].

With this retrospective study, we show that dedicated lung MRI can detect, localise and classify congenital bronchopulmonary foregut anomalies with high accuracy and very good agreement between readers. In the 39 patients, 44/46 (95.6%) abnormalities were correctly classified compared to an expert diagnosis based on pathology, surgery and/or other imaging. Only 2/39 (5.1%) lung lesions were misclassified due to misinterpretation of imaging findings: one case of bronchial atresia as cystic pulmonary malformation and one isolated overinflation as hybrid lesion. In the subgroup of our patients who underwent MRI and CT (13/39, 33.3%), the concordance rates between MRI and CT were 100% for both lesion classification and detection of systemic arteries. In the subgroup of our patients with resected lung lesions (24 patients, 28 lung lesions), the concordance rate between MRI and pathology was 92.9% for diagnosing and detecting systemic arteries. Overall, the agreement between MRI and the final diagnosis in our study was comparable to that reported for contrast-enhanced CT and pathology in recent series. Mon et al. [23] described concordance rates between CT and pathology for diagnosing congenital pulmonary airway malformation of 83.5% and detecting systemic feeding arteries of 90.2%. Narayan et al. [24] reported similar diagnoses at CT and pathology in 40/45 children with resected congenital lung lesions (concordance rate: 88.9%).

On MRI, respiratory gated T2-weighted fast spin echo sequences with radial readout allowed the differentiation between consolidation, cystic lesions filled with air or fluid, and hyperinflated lung parenchyma filled with fluid in the first days of life or with air later on. Bronchial remnants impacted with mucus were detected as linear, branching or more globular structures with high signal intensity in different types of isolated overinflation (bronchial atresia, segmental overinflation) but also in bronchopulmonary sequestrations and hybrid lesions. The presence of mucoid impaction may confirm the hypothesis that these lung lesions are related to airway obstruction with secondary pulmonary dysplastic changes and associated vascular anomalies [25].

The dynamic contrast-enhanced series helped detect bronchopulmonary lung lesions as they all presented as a perfusion defect during peak pulmonary enhancement. In addition, the dynamic series allowed the differentiation between atelectasis with increased signal intensity and consolidation as part of the lung lesion with perfusion defect at peak pulmonary enhancement. Systemic arterial supply and abnormal pulmonary venous drainage of the lung lesions could be detected on angiographic images from the dynamic contrast-enhanced series, while the evaluation of parenchymal perfusion allowed the further description of the vascular supply of pulmonary abnormalities. Lung areas of overinflation, bronchopulmonary sequestrations, hybrid lesions and normal lung parts supplied by systemic arteries showed delayed peak enhancement of about 2 to 4 s, which indicates predominantly systemic arterial supply of these lesions due to either absent pulmonary arteries or decreased pulmonary arterial perfusion as a sequelae of hypoventilation and decreased oxygen saturation. While contrast-enhanced CT may delineate small vessels comparable to MRI, the perfusion information obtained with a dynamic MRI series is novel in the investigation of congenital lung lesions.

With the clinical introduction of ultrashort echo time sequences for lung MRI, there is hope that these sequences may better delineate small cystic lesions than current techniques do. However, in our preliminary experience with four congenital lung lesions, areas of overinflation are not very conspicuous and consolidation may obscure systemic feeding arteries.

The main limitation of our study is its retrospective nature, which could have introduced a selection bias. However, we believe this selection bias to be relatively small as almost all patients with congenital lung malformations seen at our institution during the study period underwent MRI. Other limitations include the lack of surgery and pathology for confirmatory diagnosis in one-third of our patient cohort, since not all patients received surgery for their abnormality. Therefore, final expert diagnosis was based only on other imaging for these patients. This reflects the current praxis that some congenital bronchopulmonary abnormalities are not treated by surgery but managed conservatively [11, 17, 21, 26, 27]. At our centre, we follow prenatally detected lung lesions by foetal US and consider foetal MRI for prognostic purposes. After birth, we acquire lung MRI for confirmation of the lesion and for planning treatment. In asymptomatic infants, the postnatal MRI examination is usually obtained between 3 and 5 months of age, so it can be performed without general anaesthesia and used to assess the need for surgical treatment, which is usually scheduled around the age of 6 months. Large bronchopulmonary sequestrations with relevant shunt and congenital pulmonary airway malformations are generally resected due to the risk of infection [2]. We also recommend resection of multicystic lung lesions (CPAM types 1 and 2), because they cannot reliably be differentiated from cystic bronchopulmonary blastoma on gross pathology or imaging [2, 28]. Conservative management is considered for asymptomatic lesions with overinflation.

The main drawback of dedicated lung MRI is the rather long imaging time that requires sedation in small children. Although fast lung imaging is possible with single-shot fast spin echo or steady-state free precession techniques, in our experience these fast sequences alone are not sufficient for characterising parenchymal lung lesions and they do not provide sufficient information on lung vasculature or perfusion [29]. By limiting the MRI protocol to respiratory-gated fast spin echo sequences in two planes and the dynamic contrast-enhanced series, the imaging time could be reduced to approximately 10 min. Currently, we consider gadolinium-based contrast agents indispensable for MRI assessment of most congenital thoracic malformations, mainly for delineation of pathological vessels and reliable detection of lung lesions. Like many other paediatric centres, we have stopped using linear gadolinium compounds and only apply macrocyclic agents, for which deposition in tissue appears to be less or absent [30].

The choice of the imaging modality for postnatal assessment of congenital thoracic malformations will depend on the image quality achievable by the MRI and CT equipment available. If a fast multi-row detector CT scanner allowing artefact free imaging of lung parenchyma in free-breathing infants is available, one will have to weigh the risk of ionising radiation from CT against the risk of sedation and gadolinium deposition in tissues for MRI [31].

## Conclusion

Dedicated lung MRI can reliably delineate parenchymal and vascular components of lung lesions in young children. Dynamic contrast-enhanced sequences facilitate their detection and provide information on lung perfusion. Therefore, comprehensive imaging characterisation of congenital bronchopulmonary malformations has become feasible and dedicated lung MRI may be considered a radiation-free alternative to contrast-enhanced CT.
